# Long-Lasting Response Changes in Deep Cerebellar Nuclei *in vivo* Correlate With Low-Frequency Oscillations

**DOI:** 10.3389/fncel.2019.00084

**Published:** 2019-03-06

**Authors:** Letizia Moscato, Ileana Montagna, Licia De Propris, Simona Tritto, Lisa Mapelli, Egidio D’Angelo

**Affiliations:** ^1^Department of Brain and Behavioral Sciences, University of Pavia, Pavia, Italy; ^2^IRCCS Mondino Foundation, Pavia, Italy

**Keywords:** deep cerebellar nuclei, cerebellum, plasticity, oscillations, *in vivo* electrophysiology

## Abstract

The deep cerebellar nuclei (DCN) have been suggested to play a critical role in sensorimotor learning and some forms of long-term synaptic plasticity observed *in vitro* have been proposed as a possible substrate. However, till now it was not clear whether and how DCN neuron responses manifest long-lasting changes *in vivo*. Here, we have characterized DCN unit responses to tactile stimulation of the facial area in anesthetized mice and evaluated the changes induced by theta-sensory stimulation (TSS), a 4 Hz stimulation pattern that is known to induce plasticity in the cerebellar cortex *in vivo*. DCN units responded to tactile stimulation generating bursts and pauses, which reflected combinations of excitatory inputs most likely relayed by mossy fiber collaterals, inhibitory inputs relayed by Purkinje cells, and intrinsic rebound firing. Interestingly, initial bursts and pauses were often followed by stimulus-induced oscillations in the peri-stimulus time histograms (PSTH). TSS induced long-lasting changes in DCN unit responses. Spike-related potentiation and suppression (SR-P and SR-S), either in units initiating the response with bursts or pauses, were correlated with stimulus-induced oscillations. Fitting with resonant functions suggested the existence of peaks in the theta-band (burst SR-P at 9 Hz, pause SR-S at 5 Hz). Optogenetic stimulation of the cerebellar cortex altered stimulus-induced oscillations suggesting that Purkinje cells play a critical role in the circuits controlling DCN oscillations and plasticity. This observation complements those reported before on the granular and molecular layers supporting the generation of multiple distributed plasticities in the cerebellum following naturally patterned sensory entrainment. The unique dependency of DCN plasticity on circuit oscillations discloses a potential relationship between cerebellar learning and activity patterns generated in the cerebellar network.

## Introduction

Two functional aspects of the cerebellum, that have been emphasized in turn but proved hard to reconcile, are the pronounced oscillatory dynamics (Llinas, 1988) and the role in sensorimotor learning (Marr, 1969; Albus, 1971; Ito, 1972). Key nodes in the cerebellar circuitry are the deep cerebellar nuclei (DCN). DCN convey rhythmic outputs to the motor system (Jacobson et al., 2008) and, at the same time, have been suggested to be the site of plasticity by studies using local lesions (Ohyama et al., 2003, 2006) or electrical stimulation of afferent fiber bundles (Racine et al., 1986). Multiple forms of plasticity have been reported in DCN synapses *in vitro* (Morishita and Sastry, 1996; Ouardouz and Sastry, 2000; Zhang et al., 2004; Zhang and Linden, 2006; Pugh and Raman, 2009) (reviewed in Hansel et al., 2001; Gao et al., 2012; D’Angelo, 2014; Mapelli et al., 2015; D’Angelo et al., 2016b) and have been proposed to play a critical role in animal associative behaviors by computational models (Medina and Mauk, 1999; Casellato et al., 2015; Antonietti et al., 2016; D’Angelo et al., 2016a). Despite this evidence, the demonstration that long-lasting changes can actually be measured in DCN *in vivo* and can be related to internal circuit oscillations and plasticity was still lacking.

Deep cerebellar nuclei neurons are autorhythmic (Jahnsen, 1986a,b) and receive both excitatory inputs from collaterals of mossy and climbing fibers and inhibitory inputs from Purkinje cells (PCs) (Llinas and Muhlethaler, 1988). DCN neurons respond to tactile stimulation generating discharge patterns, which reflect the combination of inhibitory and excitatory inputs (Rowland and Jaeger, 2005, 2008; Chen et al., 2010; Canto et al., 2016; Yarden-Rabinowitz and Yarom, 2017). DCN neurons send output fibers to thalamus and to various precerebellar nuclei, influencing neuronal activity both in descending systems and in the cerebral cortex (Watson et al., 2014; Gao et al., 2018). Specific pathways also connect DCN with the inferior olive (Jacobson et al., 2008) and cerebellar granular layer (Ankri et al., 2015; Gao et al., 2016). These connections form the basis for reverberating loops that have been predicted to sustain rebound excitation and oscillatory cycles (Llinas and Muhlethaler, 1988; Kistler and De Zeeuw, 2003; Marshall and Lang, 2004; Hoebeek et al., 2010; Witter et al., 2013).

In the DCN, long-term synaptic plasticity [long-term potentiation and depression (LTP and LTD)] has been identified both at excitatory and inhibitory connections *in vitro*. Interestingly, excitatory plasticity depended on post-inhibitory rebound bursts (Pugh and Raman, 2006) and inhibitory plasticity required co-activation of mossy fibers (Morishita and Sastry, 1996; Ouardouz and Sastry, 2000), so that plasticity at the two synapses appears to be correlated and to require precise activation sequences.

In this work, we asked whether long-lasting changes could be induced in DCN single unit responses in anesthetized mice *in vivo* using facial theta sensory stimulation (TSS), which proved able in previous works to induce long-lasting changes in responses recorded from the cerebellum granular layer and molecular layer (Roggeri et al., 2008; Ramakrishnan et al., 2016). TSS actually induced long-lasting changes in DCN unit responses. Interestingly, these changes were uniquely correlated with the frequency of stimulus-induced oscillations, suggesting a close relationship between oscillatory dynamics and plasticity (D’Angelo and De Zeeuw, 2009; Cheron et al., 2016) reminiscent of induction schemes identified in hippocampus and neocortex (Buzsaki, 2006; Roy et al., 2014).

## Materials and Methods

Multiple single-unit recordings were performed from the fastigial nucleus of C57BL/6 mice of either sex (40.2 ± 1.8 days old; *n* = 51) under urethane anesthesia. Urethane was used as its anesthetic action is exerted through multiple weak effects (including a 10% reduction of NMDA, 18% reduction of AMPA and 23% enhancement of GABA-A receptor-mediated currents) (Hara and Harris, 2002) compared to ketamine or isoflurane, which act by powerfully blocking NMDA receptors (up to 80 and 60%, respectively; Hara and Harris, 2002) and could therefore severely compromise the induction of plasticity (Godaux et al., 1990; Muller et al., 1993; Bengtsson and Jorntell, 2007; Marquez-Ruiz and Cheron, 2012; Mawhinney et al., 2012). Moreover, urethane was successfully used before in similar recording conditions to investigate plasticity in the granular layer (Roggeri et al., 2008) and molecular layer (Ramakrishnan et al., 2016) of cerebellum.

All experimental protocols were conducted in accordance with international guidelines from the European Union Directive 2010/63/EU on the ethical use of animals and were approved by the ethical committee of Italian Ministry of Health (638/2017-PR; 7/2018-PR).

### Surgical Procedures

Mice were deeply anesthetized with intraperitoneal injections of urethane (Sigma-Aldrich). Induction (1.3 g/kg urethane dissolved in 0.9% NaCl) was followed by booster injections (10% of the induction dose) in order to stabilize anesthesia, starting 30 min after induction and repeating 3–4 times every 30 min. The level of anesthesia was monitored by evaluating the leg withdrawal after pinching and spontaneous whisking. The animal was then placed on a custom-built stereotaxic table covered with a heating plate (HP-1M: RTD/157, Physitemp Instruments, Inc., Clifton, NJ, United States). Body temperature was monitored with a rectal probe and maintained at 36°C through a feedback controller (TCAT-2LV controller, Physitemp Instruments, Inc., Clifton, NJ, United States). The mouse head was fixed over the Bregma to a metal bar connected to a pedestal anchored to the stereotaxic table. This arrangement allowed open access to the peri-oral area for air-puff stimulation. Surgery was performed to expose the cerebellar surface: local reflexes were reduced by subcutaneous application of lidocaine (0.2 ml; Astrazeneca), then the skin and muscles were removed. Craniotomy of the occipital bone (-7.8 mm AP, +0.50 mm ML from Bregma, in order to record from the fastigial nucleus) allowed to expose the cerebellar surface over the vermis. The *dura mater* was carefully removed and the surface was covered with saline (NaCl 0.9%; Sigma) to prevent drying.

### Single Unit Recordings *in vivo*

Quartz-coated platinum/tungsten fiber electrodes (1–5 MΩ) organized in a multi-electrode array (MEA) of 4 × 4, with inter-electrode distance of 100 μm (Eckhorn matrix, Thomas Recording, GmBh, Germany) were used for neuronal recordings. Recording electrodes were positioned over the vermis, ipsilateral to the air puff stimulator, and lowered perpendicularly to the surface down to a depth of 2109.1 ± 65.5 μm (*n* = 33). The electrophysiological signals were digitized at 25 kHz, using a 300–5000 Hz band-pass filter, amplified and stored using a RZ5D processor multi-channel workstation (Tucker-Davis Technologies, Alachua, FL, United States). DCN neurons were identified online by assessing recording depth, spontaneous activity, and stimulus-evoked responses. At the end of recordings, an electric lesion was made by injecting current through the recording electrode. The recording site was then confirmed by histological tissue processing (see below).

### Sensory Stimulation

**FIGURE 1:**
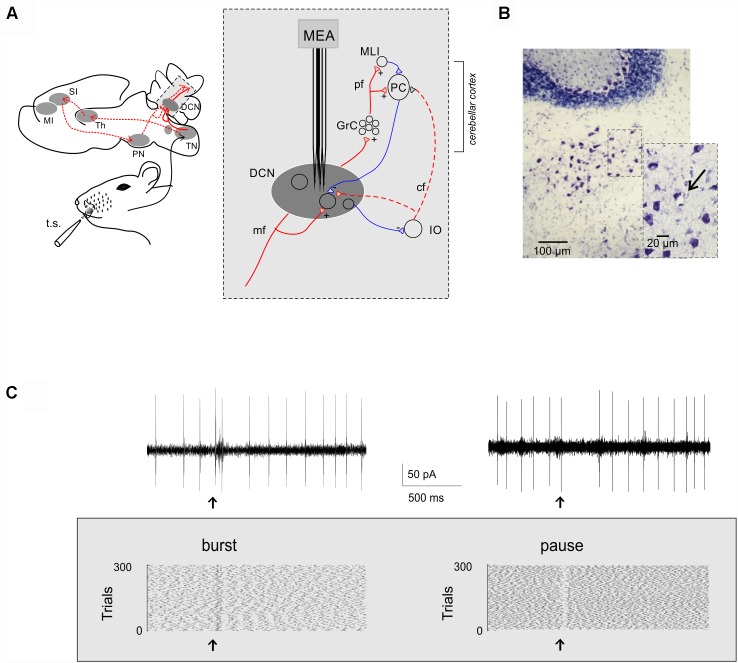
Extracellular recordings from DCN units *in vivo*. **(A)** Schematic representation of the main pathways activated by air puff stimulation of the peri-oral region in mice: the trigemino-cerebellar (*solid red line*) and thalamo-cortical-pontine (*dashed red line*) pathways. The region in the gray box is expanded at the right to show the main circuit elements relevant to DCN neuron activity. MI, primary motor cortex; SI, primary sensory cortex; Th, thalamus, PN, pontine nuclei; TN, trigeminal nucleus, DCN, deep cerebellar nuclei; GrC, granule cells; MLIs, molecular layer interneurons; PC, Purkinje cell; IO, inferior olive; pf, parallel fibers; mf, mossy fibers; cf, climbing fiber; t.s., tactile stimulation. MEA, multi-electrode array (Eckhorn matrix, see section “Materials and Methods” for details). **(B)** Toluidine blue stained coronal cerebellar slice showing the electric lesion (*arrow*) made by the recording electrode in the fastigial nucleus. **(C)** Two single-unit recordings showing a burst and a pause in response to tactile stimulation (*arrow*). The raster plots show the spike discharge during ∼2 s recordings and its change caused by tactile stimulation in 300 consecutive trials.

Tactile sensory stimulation was performed using air-puffs (30 ms pulses, 30–60 psi) delivered through a small tube ending with a nozzle (0.5 mm diameter) positioned 2–3 mm away from the snout area of the animal and connected to a MPPI-2 pressure injector (Applied Scientific Instrumentation, Eugene, OR, United States) (Roggeri et al., 2008; Ramakrishnan et al., 2016). While cerebellar cortical responses to skin receptive fields stimulation are organized in the so-called “fractured somatotopy” in the granular layer and in zonal or small regions in the molecular layer (Shambes et al., 1978; Kassel et al., 1984; Ekerot and Jorntell, 2001; Jörntell and Ekerot, 2002), DCN neurons have been described to respond to large portions of the body surface, both ipsi- and contra-lateral (Rowland and Jaeger, 2005). We nevertheless limited the sensory stimulation area to mouse upper lip, lower lip or whisker pad of the ipsilateral region. Following 5 min of spontaneous activity recording, low frequency stimuli (0.5 Hz) were delivered over the mouse upper lip, lower lip or whisker pad to activate the corresponding receptive fields and evoke the neuronal response (Bower and Woolston, 1983; Morissette and Bower, 1996; Vos et al., 1999; Roggeri et al., 2008; Ramakrishnan et al., 2016) (see Figure 1A). DCN single unit responses were monitored online by building peri-stimulus time histograms (PSTHs) triggered by the air-puffs. Once a responsive unit was detected, control stimuli were delivered for 20 min at 0.5 Hz, in order to characterize unit responses to tactile sensory stimulation. Then, the TSS pattern (a burst of 100 air-puffs at 4 Hz) was delivered, followed by post-induction recordings for at least 40 min at 0.5 Hz. Since the air puff has been reported to elicit a brief spike burst in the mossy fibers (Vos et al., 1999; Chadderton et al., 2004), the TSS is likely to determine short bursts repeated at 4 Hz. This pattern is known to induce plasticity in the cerebellar cortex (see Roggeri et al., 2008; Prestori et al., 2013; Ramakrishnan et al., 2016; Romano et al., 2018). In 12 recordings TSS was not delivered, monitoring the stability of responses for at least 60 min.

### Pharmacology

In a subset of experiments, the AMPA and NMDA receptor antagonists, 100 μM NBQX (Abcam) (Guo et al., 2016) and 250 μM D-APV (Tocris Bioscience) (Zhang et al., 2017), were injected in the fastigial nucleus near the recording electrodes. APV and NBQX were added to a Krebs solution with the following composition (in mM): 120 NaCl, 2 KCl, 1.2 MgSO_4_, 26 NaHCO_3_, 1.2 KH_2_PO_4_, 2 CaCl_2_, and 11 glucose, equilibrated with 95% O_2_–5% CO_2_ (pH 7.4). The solution containing the drugs was pre-loaded in a pneumatic picopump (PV820, World Precision Instruments), operated through adjustable air pressure, terminating in a 35G needle, positioned using a Patch-Star micromanipulator (Scientifica, Ltd.). After 15 min of single-unit recording, the solution was injected at the rate of 1 μl/5 min. It should be noted that the injection of GABAergic antagonists in the fastigial nucleus would not help discerning the origin of the pause, as it would affect the synapses coming from both local interneurons and PCs.

### Optogenetics

#### Adeno Associated Virus Injection and Expression

The expression of ChR2 in the cerebellar vermis was obtained through local injection of the adeno associated viral construct pAAV-hSyn-hChR2-EYFP (AAV1 serotype; Penn Vector Core, University of Pennsylvania, United States). C57BL6 mice of either sex (30 days old, *n* = 13) were anesthetized with 1–2% isoflurane in oxygen 100% at 0.7 L/min delivered from a gas vaporizer (Ugo Basile S.R.L., Italy) and were placed in a stereotaxic apparatus (Leica vernier stereotaxic instrument), where they constantly received isoflurane from a nose cone and had their head fixed with ear bars. Mice body temperature was constantly monitored by a heating pad connected to a rectal thermal probe (TCAT-2LV controller, Physitemp Instruments, Inc., Clifton, NJ, United States) and maintained at 36°C. After testing the absence of withdrawal reflexes, a sagittal incision on the head was performed to expose the cranium, and a burr hole was drilled to target lobule VI of cerebellar vermis 3.5 mm posterior to Lambda. The virus was loaded into a 10 μl NanoFil syringe (World Precision Instruments) that was connected to an automatic syringe pump (Ugo Basile S.R.L., Italy). The injection needle (NF35BV, 35G, World Precision Instruments) was positioned into the vermis at 300 μm depth and 0.2 μl of virus solution at a titer of 1.168^13^ genome copies/ml was injected at a flow rate of 0.05 μl/min. This procedure ensured a localized expression of ChR2 at the level of cerebellar molecular and PC layers. A good incorporation of the virus in the tissue was assured by keeping the needle in place for 10 min after the end of perfusion. The head was sutured and mice were kept under observation until recovery from the anesthesia, before returning to the animal facility. In order to ensure a good expression of ChR2, electrophysiological experiments were performed 21–28 days after viral injection.

#### ChR2 Expression in Acute Cerebellar Slices

In five mice that were injected with the construct but were not used for *in vivo* recordings, after 30 days the cerebellum was removed and used to prepare acute slices (220 μm thick) following standard procedures (Mapelli et al., 2017). The efficacy of ChR2 expression was tested by extracellular recordings from PCs. The PC soma was selectively illuminated with blue led light (Polygon400, Mightex Systems). The extracellular signals were recorded using a Multiclamp 700B amplifier (Molecular Devices) controlled by pClamp10 through a Digidata1440A (Molecular Devices). When illuminated, the PCs increased firing activity as expected from effective ChR2 expression causing membrane depolarization (Supplementary Figure S1).

#### General Aspects of Optogenetics Experiments

Since our aim was to interfere with PC activity, the site of AAV1 injection was limited to restricted regions of the molecular layer (Supplementary Figure S1A). The ChR2 was expressed under a generic neuronal promoter in common to molecular layer interneurons, PCs and parallel fibers. Since local circuit wiring in the molecular layer is not homogeneous (Valera et al., 2016), optogenetic activation was not expected to sort out the same effect in all cases (cfr. Figure 1A for a scheme of the circuit). Indeed, depending on the individual experiment, optogenetic stimulation could either increase or decrease PC activity and the pause and, in 2 out of 8 cases, no response modification was detected.

#### Light Application During *in vivo* Recordings

A light-conducting glass fiber with 120 μm diameter cladding and numerical aperture NA = 0.22 (Thomas Recording GmBh, Germany) was mounted in the Eckhorn Matrix (Thomas Recording GmBh, Germany). Just as the recording electrodes, it was possible to drive the tip of the glass fiber down into the tissue with micrometric precision. The optic fiber was connected though a FC/PC patch cable (ThorLabs Ø105 μm, 0.22NA, FC/PC-FC/PC Fiber Patch Cable, 1 m) to a 473 nm MM laser (S1FC473MM fiber coupled laser, Thorlabs) with adjustable output power (50 mW maximum). The laser was gated by a (TTL) trigger signal generated by the RZ5D bioamp processor (Tucker Davis Technologies, Alachua, FL, United States) driven by the OpenEx software controlling data acquisition. YFP fluorescence allowed to determine the effectiveness of adenoviral expression *in vivo*. The tip of the fiber was placed at about 250 μm from the surface of the cerebellum, in order to obtain a localized optical stimulation of the molecular and PC layers. Laser light pulses (50 ms) were applied at 0.5 Hz paired to the air-puff (delay of 30 ms) with a power of 0.5–1 mW. The output power, measured with a power meter (PM100D, with s130c sensor; Thorlabs) at the tip of the glass fiber, was 0.03 mW (Kruse et al., 2014).

### Histology

The location of recording electrodes in the DCN was confirmed histologically. Electrical lesions were obtained at the end of recordings by applying a 20 μA–20 s current pulse through the same recording electrode connected to a stimulus isolator and a stimulator unit. Then, the mouse was perfused transcardially with phosphate-buffered saline (PBS) followed by 4% paraformaldehyde (Sigma-Aldrich) overnight at 4°C. The fixated brains were cryo-protected with 30% sucrose solution in PBS, embedded in OCT (Cryostat embedding medium, Killik, Bio-Optica), and stored at -80°C. 20-μm-thick histological sections were obtained and stained with toluidine blue. The histological confirmation of the recording sites was obtained by microscopic observation of the stained sections (see Figure 1B).

The identification of the viral expression was also analyzed histologically from the fixated brain of injected mice. Confocal images (see Supplementary Figure S1) were taken from 20-μm-thick sections washed with PBS (three times for 5 min), counterstained with Hoechst 33258 (Thermo Fisher Scientific- 2 μg/mL) for 15 min, washed again with PBS (three times for 5 min) and finally mounted with ProLong^TM^ Gold Antifade Mountant (Thermo Fisher Scientific). Images were acquired with a TCS SP5 II (Leica Microsystems) equipped with a DM IRBE inverted microscope (Leica Microsystems) with 20, 40, or 63X objectives and visualized by LAS-AF Lite software (Leica Microsystems Application Suite Advanced Fluorescence Lite version 2.6.0) or with ImageJ (Fiji distribution, SciJava). Fluorescence microscopy showed that the site of injection was confined to limited regions of the molecular layer (see Supplementary Figure S1).

### Data Analysis and Statistics

Electrophysiological signals were acquired using OpenEx software (Tucker-Davis Technologies) and analyzed offline using custom-written routines in MATLAB (Mathworks, Natick, MA, United States) and Excel. Openscope (part of the OpenEx suite) was used to construct online PSTH triggered by air-puffs, in order to identify responding units. The raw traces were analyzed and sorted offline using SpikeTrain (Neurasmus BV, Rotterdam, Netherlands) running under MATLAB. The stability of recordings was carefully assessed ( < ± 20% amplitude fluctuation over the duration of the recording) and only units with stable spike size were considered for further analysis. PSTHs and raster plots were used for the analysis of responses to stimulation, normally consisting of peaks and pauses emerging from background discharge. To optimize PSTH resolution, a 5 ms bin width was used to analyze peaks and a 15 ms bin width was used to analyze pauses. The “burst” was defined as an increase in firing frequency generating a PSTH peak after the stimulus. The “pause” was defined as a decrease in firing frequency generating a PSTH pause after the stimulus. The threshold for peaks and pauses detection in PSTHs was set at twice the standard deviation of the basal frequency in the pre-stimulus period, calculated for each bin. No constraints on the number of bins showing significant changes compared to the pre-stimulus period were applied, since some response might show small duration (as the case of peaks, lasting 5–10 ms and therefore described by one or few bins). Statistical comparisons of peak and pause changes in optogenetic experiments was performed against changes in the stability controls at the same experimental times (histograms in Figure 3B, 4F).

The effect of TSS was evaluated by measuring the corresponding changes in PSTH peaks and pauses as the post-TSS responses (computed over the first 15 min after TSS) that exceeded twice the standard deviation of the pre-TSS response (computed over the last 15 min before TSS). Positive changes were considered as a potentiation and negative changes were considered as a suppression of basal firing (a minority of units did not show any significant changes with respect to this criterion).

Statistical comparisons were carried out using paired or unpaired Student’s *t*-test or Fisher’s *F*-test. The normality of the data was assessed using the Shapiro–Wilk test. In the few cases data were not distributed normally, the Brown-Forsythe test was applied to assess the homogeneity of variances. Data in the text are reported as mean ± SEM. Clustering *k*-mean analysis and autocorrelation analysis on PSTHs were performed using MATLAB routines. Autocorrelations were performed using a function (*xcorr*) yielding oscillation frequency and magnitude (with magnitudes normalized to 1). The statistical significance of the changes in oscillation frequency in pharmacological and optogenetic experiments was evaluated with respect to stability controls at the same experimental times.

Data fitting was performed using routines written in OriginPro8 (OriginLab, Co., Northampton, MA, United States). A Lorentzian function was used to fit the frequency-dependence of plasticity changes:

$$y=y0+\frac{2A}{\pi }\cdot \frac{w}{4{\left(x-fc\right)}^{2}+w^{2}}$$

where *y0* and *A* are curve baseline and amplitude, *w* is curve width, *fc* is the resonance frequency.

## Results

Single-unit recordings were performed from the cerebellar fastigial nucleus in urethane anesthetized mice (Figure 1A). All units were spontaneously active and showed a basal frequency of 8.19 ± 0.99 Hz (range: 2–27 Hz; *n* = 51), in agreement with previous reports of spontaneous activity under urethane anesthesia (Sweeney et al., 1992; LeDoux et al., 1998; Raman et al., 2000). The recording site was confirmed by electric lesions made through the recording electrode and identified histologically (Figure 1B). Single-unit responses to low frequency tactile stimulation (0.5 Hz) generated spike *bursts* and *pauses* modifying the basal discharge (Figure 1C) that were likely to reflect the neuronal response to excitatory and inhibitory synaptic inputs impinging onto DCN neurons (Rowland and Jaeger, 2005, 2008).

### Bursts and Pauses in DCN Unit Responses

**FIGURE 2:**
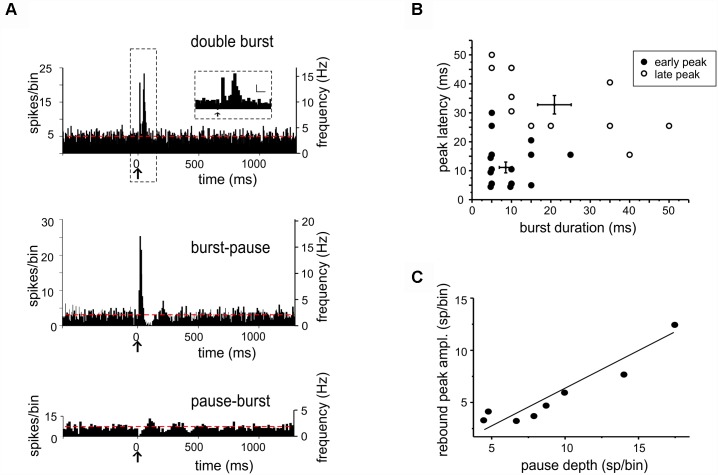
Bursts and pauses in DCN unit responses to tactile stimulation. **(A)** Example of PSTHs obtained from DCN units showing different responses to tactile stimulation (*arrows*): double burst (5 ms-bin), burst-pause (5 ms-bin), and pause-burst (15 ms-bin). Red dashed lines show the basal discharge frequency. The scale bars in the inset on top are 5 sp/bin and 25 ms. **(B)** In the pool of responses starting with a burst, two groups were discriminated using cluster analysis (*k-means*) on peak latency and burst duration. This results in the identification of early and late peaks, whose latencies are compatible with inputs from the trigeminal and cortical pathways conveying sensory stimuli to the cerebellum (cfr. Figure 1A). **(C)** Characterization of pause-burst responses. A positive correlation was found between rebound-peak amplitude and pause depth [*R*^2^ = 0.87, Fisher’s *F*-test *p(*F) = 0.001, *n* = 8].

Single-unit responses to low frequency tactile stimulation generated combinations of peaks and pauses in PSTHs and, in some cases, the response continued with an oscillation (see below). Over a total of 51 units, we identified 2 fundamental categories of patterns, with either the burst or the pause as the initial response (Figure 2A).

When the *burst* initiated the response (“burst-first” category, *n* = 26), some units (*n* = 18) showed a single PSTH peak with latency of 14.27 ± 4.07 ms (duration 11.80 ± 2.51 ms), while others (*n* = 8) showed two PSTH peaks with latencies of 9.87 ± 1.99 and 33 ± 3.7 ms (duration of 8.12 ± 1.31 and 15 ± 3.27 ms). These peak latencies corresponded to those reported for trigeminal and cortical responses of granular layer neurons (Vos et al., 1999; Roggeri et al., 2008), suggesting that the initial bursts most likely corresponded to synaptic excitation of DCN neurons through trigeminal and cortical mossy fibers (Figure 2B; see section “Materials and Methods” for details).

**FIGURE 3:**
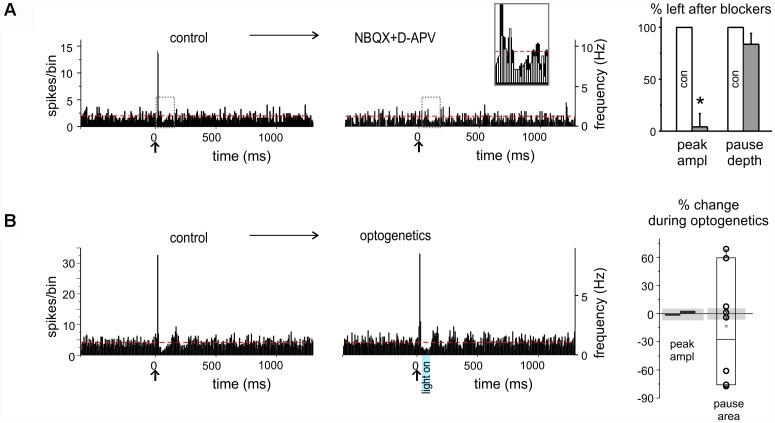
Pharmacological and optogenetic manipulations of burst and pause responses. **(A)** Example of PSTHs from a DCN unit showing a burst as initial response (*left*), that was prevented (*right*) by excitatory transmission blockers (NBQX and D-APV) injection in the nucleus (*red dashed lines* show the basal discharge frequency). The gray dashed rectangles show the areas that are overlapped in the inset. The histogram shows the % of the response, whether PSTH peaks or pauses, left after blockers injection, compared to control (*n* = 4 for both; paired Student’s *t*-test; ^∗^*p* < 0.05). **(B)** Example of PSTHs from a DCN unit showing burst-pause response before (*left*) and during (*right*) optogenetic stimulation of the molecular layer (the blue rectangle showing the time and duration of laser activation; *red dashed lines* show the basal discharge frequency). The histograms on the right show the percent change on the peak amplitude of the excitatory response and on the pause area (obtained by multiplying pause depth and duration) during optogenetic stimulation compared to control, in the single units recorded. The gray shadows show the average % change observed at the same time points in the stability controls (see section “Materials and Methods”). Note that peak amplitude is not affected, while the pause is significantly modified.

In a subset of experiments, selective AMPA and NMDA receptor antagonists (100 μM NBQX + 250 μM D-APV, respectively) were injected in the fastigial nucleus close to the recording site. In 4 (out of 4) neurons that initiated the response with a burst, the burst was abolished (peak change -95.9 ± 12.8%, *n* = 4; paired Student’s *t*-test; *p* = 0.026), while pauses remained unaltered (pause depth change -16.2 ± 10.2%; *n* = 4, paired Student’s *t*-test; *p* = 0.14; see below) (Figure 3A).

When the *pause* initiated the response (“pause-first” category, *n* = 25), it occurred with a latency of 28.8 ± 5.2 ms (duration 25.0 ± 3.8 ms). This delay was compatible with signal transmission along the mossy fiber – granule cell – PC – DCN neuronal pathway (Ramakrishnan et al., 2016), rather than resulting from local interneurons, suggesting that the initial pause most likely corresponded to DCN neuron inhibition by PCs. In a subset of experiments (*n* = 8), optogenetic stimulation of the molecular layer was applied to disrupt the cortical output, by delivering a light impulse 30 ms after the air-puff, i.e., in coincidence with the pause. In six of these recordings optogenetic stimulation caused a change in pause depth and duration exceeding three times the standard deviation of time-matched controls (see section “Materials and Methods” and histograms in Figure 3B). It should be noted that the pause in three cases increased and in three cases decreased, possibly reflecting the balance between optogenetic activation of PCs and molecular layer interneurons (see section “Materials and Methods” and Figure 1A and Supplementary Figure S1 for details). In the remaining 2 units, no evident effect of optogenetics was observed.

Several units initiating the response with a burst (21 out of 26) showed a pause following peak(s), and some units initiating the response with a pause (8 out of 24) showed a burst following the pause (Figure 2A). The nature of these *burst-pause* and *pause-burst* patterns showed peculiar properties.

**FIGURE 4:**
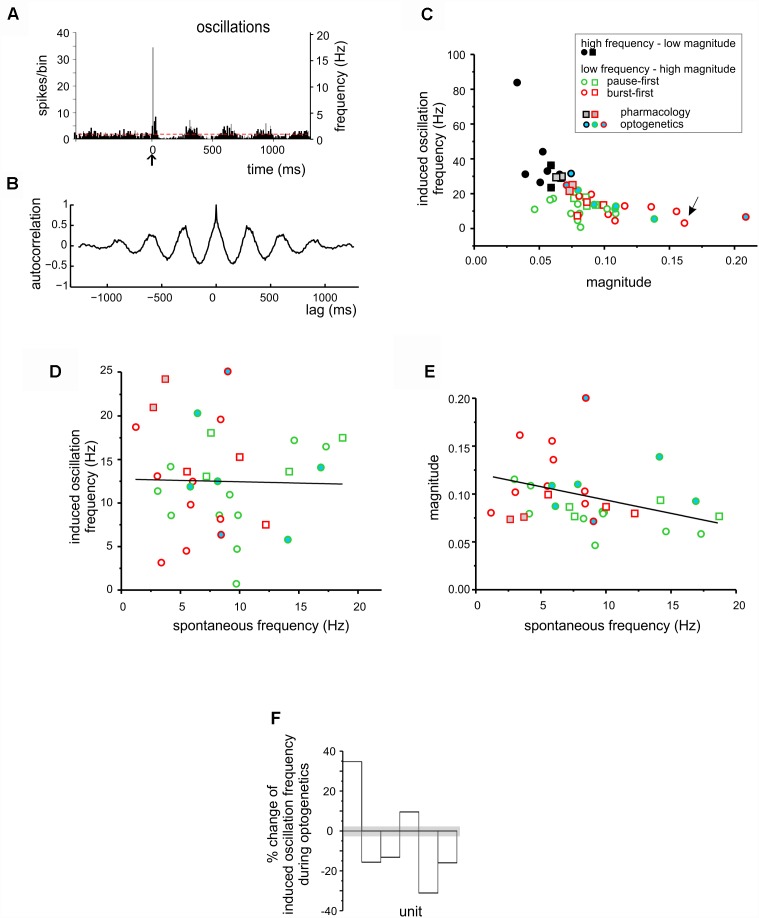
Stimulus-induced oscillations in DCN units. **(A)** PSTH obtained from a DCN unit showing low frequency oscillation following a burst-pause response. **(B)** Autocorrelogram obtained from the unit shown in **(A)** (oscillation frequency 3.15 Hz; magnitude 0.16). **(C)** The magnitude and frequency of oscillations deriving from the autocorrelation analysis shown in **(B)** were plotted for each unit. The k-means clustering revealed two groups of data, characterized by high frequency – low magnitude oscillations (black symbols) and low frequency – high magnitude oscillations (green and red symbols, for pause-first and burst-first responses respectively). The units in which the TSS was delivered are represented as circles, while those in which the TSS was not delivered are represented as squares. Gray filled symbols are used for the units in which pharmacology was applied, while blue-filled circles are used for the units in the optogenetics experiments. The arrow indicates the unit shown in **(A,B)**. **(D)** Relationship between stimulus-induced oscillation frequency and spontaneous firing frequency for the low frequency – high magnitude oscillation units in **(C)**. The linear fitting shows no evident trend [*R*^2^ = 0.03, Fisher’s *F*-test *p*(F) = 0.89]. **(E)** Relationship between magnitude of stimulus-induced oscillations and spontaneous firing frequency for the low frequency – high magnitude oscillation units in **(C)**. The linear fitting suggests a positive trend [*R*^2^ = 0.25, Fisher’s *F*-test *p*(F) < 0.08]. In **(C–E)**, the data points are divided into burst-first and pause-first units, and circles represent the units that were further used for plasticity induction (see Figure 5, 6). **(F)** The histogram shows the percent change in stimulus-induced oscillation frequency during optogenetics in the same units reported in **(C–E)**. The gray shadow shows the average percent change observed at the same time points in the stability controls (see section “Materials and Methods”).

In *burst-pause* responses, the pause was significantly delayed (58.17 ± 5.52 ms, *n* = 21, unpaired Student’s *t*-test; *p* = 0.00039) compared to that measured when it initiated the response. This longer delay suggests the intervention of additional mechanisms, like signal reentry through the recently discovered DCN – granule cells connections (Gao et al., 2016) or through precerebellar nuclei (Kistler and De Zeeuw, 2003), capable of protracting and enhancing PCs activation through cerebello-cortical loops. Phase reset, an intrinsic electroresponsive phenomenon observed in neurons (e.g., see Solinas et al., 2007), was unlikely to be responsible for this effect, as explained below (see Figure 4D).

In *pause-burst* responses, the bursts followed with a latency of 90.61 ± 20.63 ms (duration 12.14 ± 3.54 ms, unpaired Student’s *t*-test; *p* = 0.01), that was significantly longer compared to that measured when it initiated the response. A positive correlation was found between pause depth and the subsequent peak amplitude [*R*^2^ = 0.89, Fisher’s *F*-test *p*(F) < 0.001, *n* = 8] (Figure 2C). A plausible explanation is that these bursts are non-synaptic and reflect post-inhibitory rebound discharge in DCN neurons (Alviña et al., 2008; Witter et al., 2013; Canto et al., 2016), which is the stronger the deeper the pause. This is supported by a recording in which the AMPA and NMDA receptor antagonists were injected in the fastigial nucleus while recording a pause-burst unit. In this case, the burst following the pause was unaffected (single observation, not shown).

### Spontaneous Activity and Stimulus-Induced Oscillations in DCN Units

The PSTH elicited by tactile stimulation in several cases showed an *oscillation* following the initial peaks and pauses (Figure 4A). This oscillatory pattern was apparent in autocorrelation analysis (Figure 4B). The frequency/magnitude plot revealed a negative trend, with slower oscillations showing larger magnitude and *vice versa* (Figure 4C). K-means analysis identified two significantly different clusters of points (unpaired Student’s *t*-test; *p* = 0.00175), one at higher and the other at lower frequency. Low-frequency oscillations averaged 12.7 ± 1.0 Hz, *n* = 34.

The relationship between low-frequency stimulus-induced oscillations and spontaneous activity is shown in Figure 4D,E. No significant correlation was found either for frequency [*R*^2^ = 0.03, Fisher’s *F*-test *p*(F) = 0.89, *n* = 34] or magnitude [*R*^2^ = 0.25, Fisher’s *F*-test *p*(F) < 0.08, *n* = 34]. It should be noted that, out of 34 units, 15 were of the burst-first and 19 of the pause-first category. At a closer analysis, the burst-first units showed a significantly higher magnitude (0.11 ± 0.01 vs. 0.08 ± 0.01; unpaired Student’s *t*-test; *p* = 0.037) and lower spontaneous frequency (6.2 ± 0.8 vs. 10.0 ± 1.1 Hz, unpaired Student’s *t*-test; *p* = 0.01) than the pause-first units (Figure 4C–E) suggesting the existence of two distinct functional classes of DCN neurons (see below).

The injection of AMPA and NMDA receptor antagonists in the fastigial nucleus did not modify the stimulus induced oscillation frequency of the units (average absolute variation from control of 5.1 ± 0.6%, not different from that of stability controls of 5.1 ± 0.5%, *n* = 4 and *n* = 10 respectively; unpaired Student’s *t*-test; *p* = 0.99). Conversely, optogenetic stimulation of the molecular layer caused a change in the stimulus induced oscillation frequency of the recorded units exceeding three times the standard deviation of time-matched controls (see section “Materials and Methods” and Figure 4F). It should be noted that the induced-oscillation frequency in two cases increased and in four cases decreased, possibly reflecting the balance between optogenetic activation of PCs and molecular layer interneurons.

### Long-Lasting Changes Induced by TSS in DCN Unit Responses

The cerebellar cortex in rodents is known to respond to TSS of the whisker pad with long-lasting changes in the granular and molecular layers (Roggeri et al., 2008; Diwakar et al., 2011; Prestori et al., 2013; Ramakrishnan et al., 2016). We therefore investigated whether the delivery of the same TSS pattern was able to affect DCN neuron responsiveness. We defined Spike-Related Potentiation (SR-P) and Spike-Related Suppression (SR-S) as the increase or decrease in spike response probability with respect to baseline (cf. Ramakrishnan et al., 2016), both in bursts and pauses. For simplicity, we considered only the initial bursts and pauses, since their amplitude is not influenced by preceding electrical events. The values of changes were measured for each unit in the first 15 min following TSS with respect to the last 15 min before TSS.

#### TSS-Induced Changes in Initial Bursts

**FIGURE 5:**
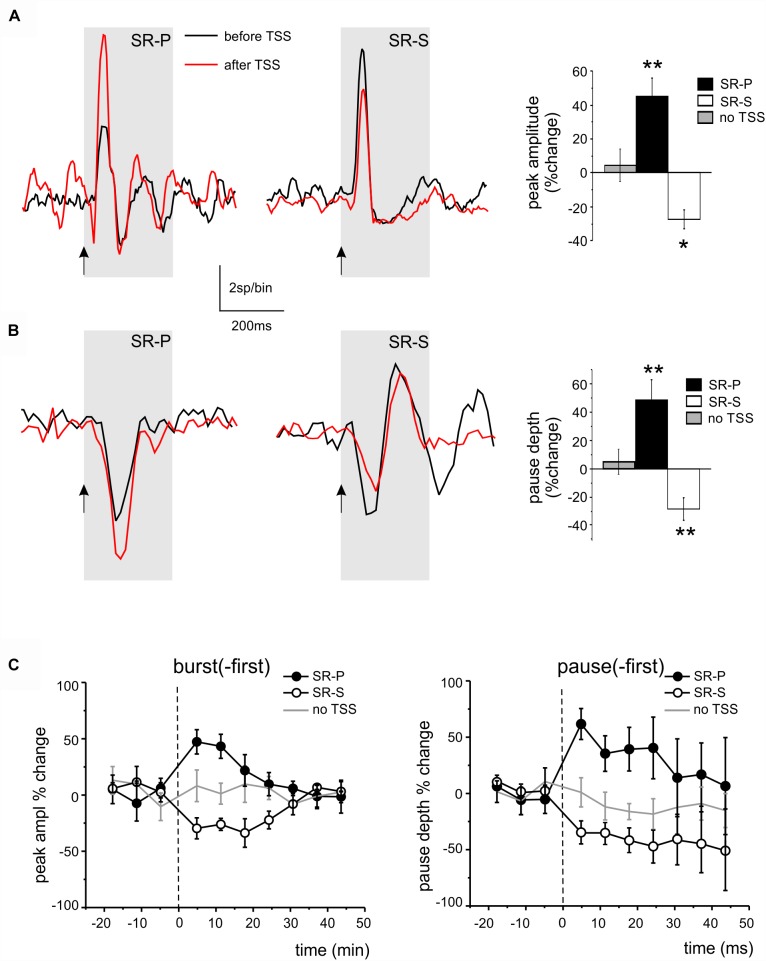
Long-lasting changes induced by TSS in DCN unit responses. **(A)** Example of PSTHs illustrating the *peak* changes (SR-P or SR-S) induced by TSS in DCN units of the burst-first category. The histogram shows the average percent changes in PSTH peak amplitude for all the units showing SR-P, SR-S, or stability controls (no TSS; paired Student’s *t*-test; ^∗^*p* < 0.05, ^∗∗^*p* < 0.01). **(B)** Example of PSTHs illustrating the *pause* changes (SR-P or SR-S) induced by TSS in DCN units of the pause-first category. The histogram shows the average percent changes in PSTH peak amplitude for all the units showing SR-P, SR-S, or stability controls (no TSS; paired Student’s *t*-test; ^∗∗^*p* < 0.01). **(C)** Average time-course of peak (in burst-first units, *left*) and pause (in pause-first units, *right*) amplitude percent changes normalized to the control period before TSS (*dashed line*) in the units showing SR-P, SR-S and in the stability group (TSS not delivered). Note that the response changes for peaks and pauses differed significantly from the stability controls in the first 15 min after the TSS.

Theta-sensory stimulation was delivered in 13 recordings showing an initial excitatory burst (burst-first, Figure 5A). A significant SR-P of the first PSTH peak was observed in four units (45.27 ± 10.73%, *n* = 4, paired Student’s *t*-test; *p* = 0.01; Figure 5A), while a significant SR-S was observed in another 8 units (-27.16 ± 5.34%, paired Student’s *t*-test; *p* = 0.01). Only in 1 out of these 13 units, no significant changes were observed.

#### TSS-Induced Changes in Initial Pauses

Theta-sensory stimulation was delivered in 13 recordings showing an initial pause in the response (pause-first, Figure 5B). A significant SR-P of the pause was observed in 4 units (48.43 ± 14.77%, *n* = 4, paired Student’s *t*-test; *p* = 0.004), while a significant SR-S of the pause depth was observed in another 7 units (-27.19 ± 7.65%, *n* = 7, paired Student’s *t*-test; *p* = 0.007; Figure 5B). In 2 units, no pause depth changes were found.

#### Stability Controls

In 7 units showing an initial excitatory burst and in 5 units showing an initial pause, TSS was not delivered. In these units, the bursts and pauses remained stable for a duration similar to that of experiments in which the TSS was delivered (bursts: 4.7 ± 11.7% change, paired Student’s *t*-test, *n* = 7, *p* = 0.9; pauses: 5.6 ± 10.0% change, *n* = 5, paired Student’s *t*-test; *p* = 0.1). These controls ruled out possible spurious changes due to intrinsic response amplitude fluctuations with time.

#### Average Time Course

The average time course for burst and pause changes was constructed by grouping all the units in each given category. The SR-P and SR-S of peaks and pauses reported above, which were statistically significant for the first 15 min after TSS, returned back to baseline within 30 min (Figure 5C).

### Correlation Between Long-Lasting Changes and Stimulus-Induced Oscillations

**FIGURE 6:**
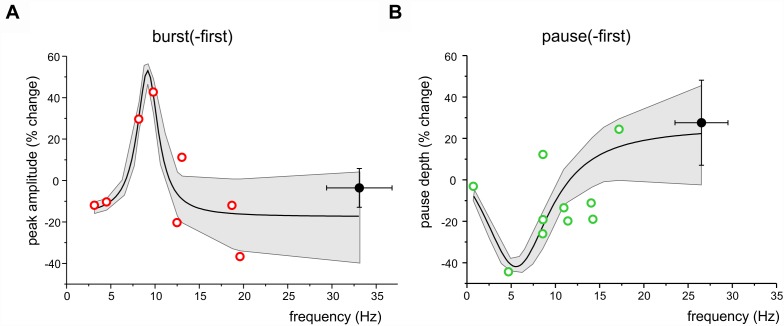
Frequency-dependence of long-lasting changes after TSS. **(A)** The plot shows the distribution of peak amplitude changes after TSS in burst-first units with respect to stimulus induced oscillation frequency. The Lorentzian fitting [*R*^2^ = 0.83; Fisher’s *F*-test *p*(F) = 0.01] shows a peak at 9.2 Hz. **(B)** The plot shows the distribution of pause amplitude changes after TSS in pause-first units with respect to stimulus induced oscillation frequency. The Lorentzian fitting [*R*^2^ = 0.78; Fisher’s *F*-test *p*(F) = 0.02] shows a peak at 5.5 Hz. Both in **(A,B)**, open symbols identify the same low-frequency units reported in Figure 4 and filled symbols are the average values ( ± SEM) of high frequency oscillation units. Both in **(A,B)**, the gray area shows the 95% confidence interval of the fitting.

In Figure 4, two functional classes of units have been identified based on their response pattern and low frequency oscillatory properties, summing up to a total of 15 burst-first units and 17 pause-first units. Here we have considered the relationship between long-lasting changes and stimulus-induced oscillation frequency in the 8 burst-first and 10 pause-first units that received TSS in control condition (without pharmacological manipulation or optogenetics). The changes were weakly correlated to frequency, with larger changes occurring at lower frequencies [linear correlation: *R*^2^ = -0.07 and *p*(F) < 0.05 for *burst-first* units; *R*^2^ = 0.32 *p*(F) < 0.03 for *pause-first* units]. The hypothesis that changes were centered on the theta-band was assessed by fitting the data using resonant functions. In particular, the Lorentzian distribution fitted the data better than linear, suggesting that peak and pause changes after TSS might be correlated with the frequency of stimulus-induced oscillations. By using a Lorentzian distribution, peak changes in the *burst-first* units (Figure 6A) peaked at 9.2 Hz with 53% SR-P and settled down to -17.2% SR-S at lower and higher frequencies [*R*^2^ = 0.83; Fisher’s *F*-test *p*(F) < 0.01]. Pause changes in the *pause-first* units (Figure 6B) peaked at 5.5 Hz with -42% SR-S and settled to 26.2% SR-P at higher frequencies [*R*^2^ = 0.78; Fisher’s *F*-test *p*(F) < 0.02]. Thus, Lorentzian fitting of SR-P and SR-S distributions showed opposite changes in burst-first and pause-first units with peaks in the low frequency range of stimulus-induced oscillations.

**FIGURE 7:**
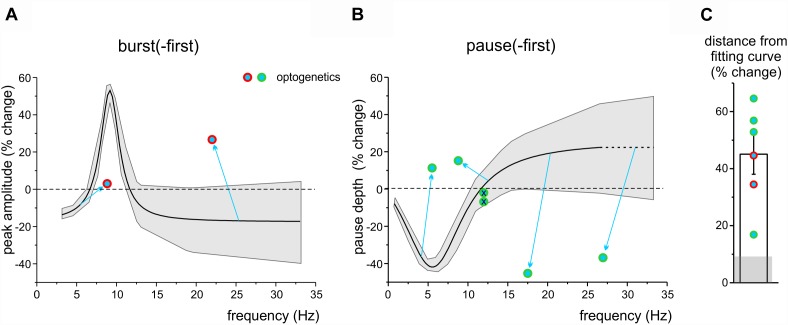
The impact of optogenetic stimulation on long-lasting response changes. **(A)** The plot shows the Lorentzian fitting as Figure 6A, with the gray area showing the 95% confidence interval. Note that the data-points representing burst-first units in which the TSS was paired with optogenetics fall far outside the confidence interval. **(B)** The plot shows the Lorentzian fitting as in Figure 6B, with the gray area showing the 95% confidence interval, extrapolated beyond the last point of control to compare new data. Note that the data-points representing pause-first units in which the TSS was paired with optogenetics fall far outside the confidence interval (except for the two points representing the units in which optogenetics did not show any effect; *crossed circles*). **(C)** The histogram shows the average distance of the optogenetics data points from the fitting curves in **(A,B)**. The gray shadow shows the average distance of control data in Figure 6 from the same fitting curves.

We then asked whether and how these long-lasting changes were influenced by cerebellar cortical activity. To this end, we used optogenetic stimulation of the cerebellar molecular layer, which allows a broader activation than electrical stimulation and is therefore more likely to capture neuronal chains involved in controlling the recorded DCN units. We have shown above that optogenetic stimulation of the molecular layer could indeed modify DCN responsiveness (see Figure 3B, 4F), disrupting the cortical output by modifying PC firing. It should be noted that, as explained in Section “Materials and Methods,” this test was not expected to yield a deterministic increase or decrease in PC firing, but rather to impact on DCN units and change their ability to generate long-term response changes after TSS. We thus compared DCN units response changes with or without the use of optogenetics assuming the Lorentzian distribution as the best fit to our data. Optogenetic stimulation of the molecular layer during TSS altered the long-lasting changes compared to those expected from controls, in such a way that these always fell beyond the confidence limits predicted from control data, both for peaks in burst-first units (Figure 7A) and for pauses in pause-first units (Figure 7B). The distance from the control curves in Figure 6A,B, estimated at the frequency of stimulus-induced oscillation recorded during optogenetic stimulation, was 45.1 ± 7.0% (*n* = 6, unpaired Student’s *t*-test; *p* = 0.03; significantly different from the distance from the same curve calculated from control data: 9.1 ± 2.1, *n* = 20, unpaired Student’s *t*-test; *p* = 0.0028; Figure 7C). Therefore, optogenetics did not seem to primarily address the same mechanism of frequency-dependent induction of long-lasting changes occurring in DCN units but rather to affect different mechanisms, presumably located in the molecular layer (see section “Discussion”).

## Discussion

Deep cerebellar nuclei units were spontaneously active and responded to tactile sensory stimulation with different combinations of bursts, pauses and oscillations. Following theta-frequency stimulation (TSS), DCN units showed spike-related potentiation or suppression, SR-P or SR-S, both in bursts and pauses. To our knowledge, SR-P and SR-S are the first electrophysiological evidence that long-lasting changes can be observed following naturally patterned sensory entrainment in DCN neurons *in vivo*. Unique in the cerebellum among the other long-lasting changes observed *in vivo* (Roggeri et al., 2008; Gao et al., 2012; D’Angelo, 2014; D’Angelo et al., 2016a; Ramakrishnan et al., 2016), the DCN SR-P/SR-S distributions were correlated to the stimulus-induced oscillation frequency of DCN units through Lorentzian functions peaking in the theta-frequency range, disclosing the complex nature of the underlying plasticity mechanisms.

### The Nature of DCN Unit Responses

All DCN units responded to tactile stimulation with short delays typical of the fast cerebellar reaction to sensory inputs. In some units (53%), bursts were the first DCN response and occurred either as a single peak at 10–14 ms or a double peak (in a third of cases) about 10 ms later. This pattern closely matches that observed in the granular layer (Morissette and Bower, 1996; Vos et al., 1999; Roggeri et al., 2008), suggesting that DCN neurons can receive double mossy fiber activation through the trigeminal pathway and the somato-sensory cortex [the trigeminal connection might not be direct for the fastigial nucleus though (Morcuende et al., 2002; Rowland and Jaeger, 2005, 2008)]. The excitatory nature of these bursts was confirmed by their extinction after injection of AMPA and NMDA receptors blockers into the DCN. In the remaining units (47%), pauses were the first DCN response with delays of 25–29 ms, most likely reflecting signal transfer through the cerebellar cortex down to PCs and DCN. This delay can be accounted for by considering that PC excitation through mossy fibers and granule cells takes about 15 ms (Ramakrishnan et al., 2016) and an additional time is required to inhibit DCN cells. Indeed, optogenetic stimulation of the molecular layer was able to modulate pause duration and depth.

It should be noted that, in principle, climbing fibers could also contribute to DCN excitation through axonal collaterals. However, in comparable recording conditions (Ramakrishnan et al., 2016), PC complex spikes that reflect climbing fiber activation were only sporadically observed and had a latency of 40–50 ms, which is too long to explain the latency of the DCN unit responses. Although climbing fibers could contribute to DCN activation, when actively stimulated (Mogensen et al., 2017), it seems very unlikely that they took part to generate the PSTH peaks analyzed here.

The pauses occurring after initial bursts were also modified by optogenetic stimulation of the molecular layer and reflected therefore DCN inhibition by PCs. These pauses, which were evident about 50 ms after the stimulus, could have been protracted by signal reentry into the cerebellar cortex through intracerebellar (Ankri et al., 2015; Gao et al., 2016) or extracerebellar loops (Kistler and De Zeeuw, 2003). The bursts following the pauses correlated with the depth of the preceding pause and were therefore probably rebound activities due to intrinsic electroresponsiveness (Alviña et al., 2008; Hoebeek et al., 2010; Witter et al., 2013) (this conclusion was supported by rebound burst persistence after injection of AMPA and NMDA receptors blockers into the DCN in a single experiment).

Some units continued their response with an oscillatory cycle independent on whether the responses started with a burst or a pause. The frequency and magnitude of oscillations induced by stimulation were not significantly correlated with spontaneous discharge in the same units. Therefore, oscillations could not be explained by phase reset, since in that case the two frequencies should coincide (Solinas et al., 2007). The origin of these oscillations should then reflect circuit mechanisms (see Figure 1A). A first mechanism was hypothesized by Yarom (Jacobson et al., 2008; Chen et al., 2010) and involves DCN control of inferior olive (IO) oscillations that reverberate through climbing fibers into the DCN-PC-IO loop. Stimulus-induced oscillations similar to ours are indeed evident in the PSTH of DCN neurons following direct climbing fiber activation (Cheron and Cheron, 2018). A second mechanism hypothesized by De Zeeuw could involve signal reentry through extra-cerebellar circuits (Kistler and De Zeeuw, 2003; Gao et al., 2016) or through the more recently identified connections between DCN and granular layer (Ankri et al., 2015; Gao et al., 2016), therefore passing again through PCs. That PCs could actually be a node in the loops controlling the stimulus-induced oscillations was supported by their perturbation by optogenetic stimulation of the molecular layer.

There were two groups of units showing stimulus-induced ocillations, *burst-first* and *pause-first* units, which turned out to show opposite frequency-dependent changes following TSS. The potential relationship between these functional groups and the DCN neuron subpopulations reported *in vitro* (Bagnall et al., 2009; Uusisaari and Knopfel, 2012) remains to be determined.

### Long-Lasting Changes in DCN Unit Responses and Their Relationship With Plastic Mechanisms

By being connected to the sensory input through multi-synaptic chains, the long-lasting changes in DCN unit responses could either be generated locally or occur upstream in the cerebellar cortex.

On one hand, according to fittings using Lorentzian functions, SR-P peaked at 9.2 Hz in *burst-first* units and SR-S at 5.5 Hz in *pause-first* units. This property favors the engagement of local mechanisms, since no similar frequency-dependent changes have ever been observed either in the granular or molecular layer *in vivo* (Roggeri et al., 2008; Ramakrishnan et al., 2016) or *in vitro* (for review see Hansel et al., 2001; Gao et al., 2012; D’Angelo, 2014; D’Angelo et al., 2016b). Moreover, LTP and LTD reported in DCN neurons *in vitro* are based on specific sequences of excitation, inhibition and rebounds (Morishita and Sastry, 1996; Ouardouz and Sastry, 2000; Pugh and Raman, 2006; Zhang and Linden, 2006). Therefore, the different synapses impinging on DCN neurons could reciprocally influence one each other, providing a plausible mechanism for SR-P and SR-S induction during stimulus-induced oscillations. The robust potentiation in PC responses observed *in vivo* following TSS (Ramakrishnan et al., 2016) is likely to contribute to DCN plasticity in *pause-first* units, independently of the frequency of stimulus-induced oscillations.

On the other hand, SR-P and SR-S of the initial burst and pause were remarkably altered by optogenetic stimulation of the molecular layer. Optogenetic stimulation caused long-lasting changes in DCN unit responses that went much beyond those expected from the alteration of stimulus-dependent oscillation frequency. This suggests the engagement of additional mechanisms. For example, similar to electrical stimulation, optogenetic stimulation might cause a broad set of long-lasting changes in the molecular layer (for details see, Ramakrishnan et al., 2016) modifying the PC output, which would eventually perturb the long-lasting changes observed in DCN unit responses. Further insights on the role of cortical input on DCN responses to TSS might derive from the use of genetically modified models with known alterations in the cerebellar cortex (e.g., mice lacking the phosphatase PP2B in PCs, that show selective loss of PC potentiation, as in Schonewille et al., 2010; Romano et al., 2018).

A further issue is about the time course of SR-P and SR-S, which decayed over 30 min. Both in rodents and humans, cerebellar learning has been predicted to occur in two steps, a faster one in the cerebellar cortex and a slower one in DCN (Medina and Mauk, 2000; Attwell et al., 2001; Smith et al., 2006; Monaco et al., 2014). Mathematical modeling further predicts that fast plasticity at the parallel fiber-PC synapse would be able to tune slow and more stable plasticity in DCN (Medina et al., 2001; Garrido et al., 2013; Casellato et al., 2014). So why *in vivo* recordings have shown more persistent changes in the granular layer (Roggeri et al., 2008) and molecular layer (Ramakrishnan et al., 2016) than in DCN? There are three key issues to consider. First, more intense or repeated stimulation may be needed to promote plasticity consolidation in DCN. Secondly, here DCN was not entrained in active sensorimotor feedback that can enhance cerebellar oscillations (Marshall and Lang, 2004). Thirdly, there was no attentional or motivational state, as the animal was anesthetized. Indeed, neuromodulation by noradrenaline, acetylcholine or serotonin is thought to be critical to drive oscillations and plasticity and promote learning (Sugihara et al., 1995; Schweighofer et al., 2004). It should also be noted that a similar trend, with a stimulus inducing long-term plasticity in the cerebellar cortex but having less effects on the cerebellar nuclei, has been recently described for the anterior interposed nucleus in cats (Mogensen et al., 2017).

It cannot be excluded that stimulus-induced oscillations and the induction or expression of long-lasting changes in DCN neuron responses might have been influenced by anesthesia. We notice, however, that urethane is very conservative on the NMDA and GABA-A receptor-dependent mechanisms of neurotransmission and has been successfully used to demonstrate long-lasting changes at other cerebellar synapses *in vivo* (Roggeri et al., 2008; Ramakrishnan et al., 2016). Moreover, stimulus-induced oscillations in DCN neurons have been recently shown using ketamine-xylidodihydrothiazin anesthesia (Cheron and Cheron, 2018).

## Conclusion

The identification of a possible relationship between DCN long-lasting changes and oscillatory dynamics engaged by tactile stimuli suggests that two key cerebellar functions can be reconciled: oscillatory activity in DCN may not just be needed to gate motor activity (Llinas, 1988; Marshall and Lang, 2004) but also to control plasticity and acquisition of sensorimotor engrams (D’Angelo and De Zeeuw, 2009; Cheron et al., 2016). Interestingly, the preferential frequency of long-lasting DCN response changes identified by Lorentzian fittings was in the theta-band, i.e., the characteristic oscillatory frequency of the IO-PC-DCN circuit (Jacobson et al., 2008) and of the cerebello-extracerebellar loops (Kistler and De Zeeuw, 2003) following mossy fiber inputs. It is tempting to speculate that the cerebellum uses oscillating and resonant mechanisms similar to those that are known to favor the induction of plasticity in hippocampal and cortical synapses (Buzsaki, 2006; Roy et al., 2014) and that the frequency of oscillations provides a signal binding DCN plasticity to specific neuronal ensembles and brain states (Buzsaki, 2005; Buzsaki, 2006; Timofeev, 2011). This would be eventually reflected into neuro-muscular coherence on the systemic scale (Gruart et al., 2000; Koekkoek et al., 2002; Sánchez-Campusano et al., 2007, 2009; Wang et al., 2018). The frequency dependence of burst and pause changes in DCN units *in vivo* prompts for a further characterization of LTP and LTD mechanisms in DCN neurons *in vitro*, also considering the existence of functionally distinct DCN neuronal populations. New experiments may also be conducted in awake animals and combined with computational modeling (Medina and Mauk, 2000; Casellato et al., 2014; Luque et al., 2014; D’Angelo et al., 2016a) to address the impact of frequency-dependent forms of plasticity during cerebellar adaptation and learning.

## Data Availability

The datasets generated for this study are available on request to the corresponding author.

## Author Contributions

LeM performed *in vivo* recordings, data analysis, and wrote the first draft of the manuscript. IM performed the sets of *in vivo* recordings with pharmacology and optogenetics, and data analysis. LDP performed the initial *in vivo* recordings. ST performed histology and image analysis. LiM and ED coordinated the work and wrote the manuscript. All authors approved the final version of the manuscript.

## Conflict of Interest Statement

The authors declare that the research was conducted in the absence of any commercial or financial relationships that could be construed as a potential conflict of interest.

## Supplementary Material

Figure S1 ChR2 expression in the cerebellar cortex of mice used for optogenetics. **(A)** Confocal image of green fluorescence (YFP) in the cerebellar cortex of a mouse sacrificed after *in vivo* recordings. Since YFP is expressed under the same promoter, YFP fluorescence reports the place of ChR2 expression. Note that fluorescence is mostly confined to the molecular and Purkinje cell layers. GL, granular layer; ML, molecular layer; PC, Purkinje cell. **(B)** Acute brain slice in bright field, with the recording electrode placed near a Purkinje cell (PC). The blue circle indicates that illuminated region is confined to PC soma and the trace shows the corresponding response in the stimulated PC (single trace, scale bars: 10 pA/200 ms). Note the increase in PC firing frequency during optical stimulation, followed by a pause configuring a typical burst-pause PC response (Cao et al., 2012; Herzfeld et al., 2015; Masoli and D’Angelo, 2017). GL, granular layer; ML, molecular layer.
