# Corrigendum: Long-Lasting Response Changes in Deep Cerebellar Nuclei *in vivo* Correlate With Low-Frequency Oscillations

**DOI:** 10.3389/fncel.2019.00313

**Published:** 2019-07-10

**Authors:** Letizia Moscato, Ileana Montagna, Licia De Propris, Simona Tritto, Lisa Mapelli, Egidio D'Angelo

**Affiliations:** ^1^Department of Brain and Behavioral Sciences, University of Pavia, Pavia, Italy; ^2^IRCCS Mondino Foundation, Pavia, Italy

**Keywords:** deep cerebellar nuclei, cerebellum, plasticity, oscillations, *in vivo* electrophysiology

In the published article, there was an error in affiliation **2**. Instead of “**Brain Connectivity Center, C. Mondino National Neurological Institute, Pavia, Italy**,” it should be “**IRCCS Mondino Foundation, Pavia, Italy**.” The authors apologize for this error and state that this does not change the scientific conclusions of the article in any way. The original article has been updated.

