# Supplementary figures and images for: Long-Lasting Response Changes in Deep Cerebellar Nuclei in vivo Correlate With Low-Frequency Oscillations
Source: Front Cell Neurosci. 2019 Mar 6;13:84. doi: 10.3389/fncel.2019.00084 (PMC6414422; doi:10.3389/fncel.2019.00084)

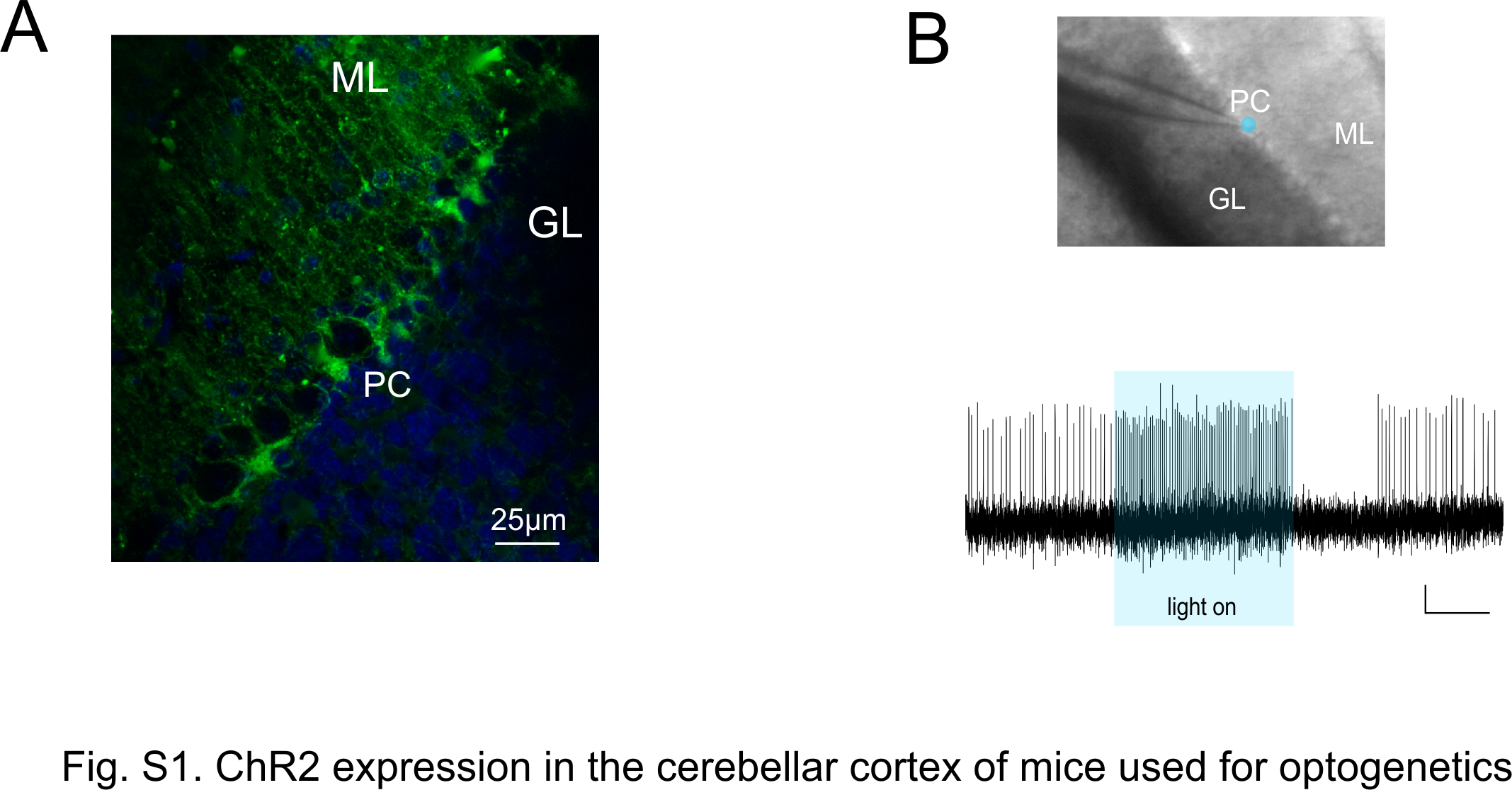

Supplement: Figure S1 — ChR2 expression in the cerebellar cortex of mice used for optogenetics. (A) Confocal image of green fluorescence (YFP) in the cerebellar cortex of a mouse sacrificed after in vivo recordings. Since YFP is expressed under the same promoter, YFP fluorescence reports the place of ChR2 expression. Note that fluorescence is mostly confined to the molecular and Purkinje cell layers. GL, granular layer; ML, molecular layer; PC, Purkinje cell. (B) Acute brain slice in bright field, with the recording electrode placed near a Purkinje cell (PC). The blue circle indicates that illuminated region is confined to PC soma and the trace shows the corresponding response in the stimulated PC (single trace, scale bars: 10 pA/200 ms). Note the increase in PC firing frequency during optical stimulation, followed by a pause configuring a typical burst-pause PC response (Cao et al., 2012; Herzfeld et al., 2015; Masoli and D’Angelo, 2017). GL, granular layer; ML, molecular layer. [file Image_1.TIF]
